# Llama 3.1 405B Is Comparable to GPT-4 for Extraction of Data from Thrombectomy Reports—A Step Towards Secure Data Extraction

**DOI:** 10.1007/s00062-025-01500-z

**Published:** 2025-02-25

**Authors:** Nils C. Lehnen, Johannes Kürsch, Barbara D. Wichtmann, Moritz Wolter, Zeynep Bendella, Felix J. Bode, Hanna Zimmermann, Alexander Radbruch, Philipp Vollmuth, Franziska Dorn

**Affiliations:** 1 Department of Neuroradiology, University Hospital Bonn, Rheinische Friedrich-Wilhelms-Universität Bonn, Venusberg-Campus 1, 53127 Bonn, Germany; 2 High Performance Computing & Analytics Lab, Rheinische Friedrich-Wilhelms-Universität Bonn, Bonn, Germany; 3 Department of Vascular Neurology, University Hospital Bonn, Rheinische Friedrich-Wilhelms-Universität Bonn, 53127 Bonn, Germany; 4 Institute of Neuroradiology, University Hospital, LMU Munich, Munich, Germany

**Keywords:** Mechanical thrombectomy, Ischemic stroke, Large language model, Llama 3, Llama 3.1, Mixtral 8X7B

## Abstract

**Purpose:**

GPT‑4 has been shown to correctly extract procedural details from free-text reports on mechanical thrombectomy. However, GPT may not be suitable for analyzing reports containing personal data. The purpose of this study was to evaluate the ability of the large language models (LLM) Llama3.1 405B, Llama3 70B, Llama3 8B, and Mixtral 8X7B, that can be operated offline, to extract procedural details from free-text reports on mechanical thrombectomies.

**Methods:**

Free-text reports on mechanical thrombectomy from two institutions were included. A detailed prompt was used in German and English languages. The ability of the LLMs to extract procedural data was compared to GPT‑4 using McNemar’s test. The manual data entries made by an interventional neuroradiologist served as the reference standard.

**Results:**

100 reports from institution 1 (mean age 74.7 ± 13.2 years; 53 females) and 30 reports from institution 2 (mean age 72.7 ± 13.5 years; 18 males) were included. Llama 3.1 405B extracted 2619 of 2800 data points correctly (93.5% [95%CI: 92.6%, 94.4%], *p* = 0.39 vs. GPT-4). Llama3 70B with the English prompt extracted 2537 data points correctly (90.6% [95%CI: 89.5%, 91.7%], *p* < 0.001 vs. GPT-4), and 2471 (88.2% [95%CI: 87.0%, 89.4%], *p* < 0.001 vs. GPT-4) with the German prompt. Llama 3 8B extracted 2314 data points correctly (86.1% [95%CI: 84.8%, 87.4%], *p* < 0.001 vs. GPT-4), and Mixtral 8X7B extracted 2411 (86.1% [95%CI: 84.8%, 87.4%], *p* < 0.001 vs. GPT-4) correctly.

**Conclusion:**

Llama 3.1 405B was equal to GPT‑4 for data extraction from free-text reports on mechanical thrombectomies and may represent a data secure alternative, when operated locally.

**Supplementary Information:**

The online version of this article (10.1007/s00062-025-01500-z) contains supplementary material, which is available to authorized users.

## Introduction

Mechanical thrombectomy has become the standard therapy for patients with ischemic stroke and large vessel occlusion [1–14]. Standard procedural details like door-to-groin time, Alberta Stroke Program Early CT Score (ASPECTS) and modified thrombolysis in cerebral ischemia (mTICI) are documented in the procedure reports and are collected for clinical and scientific purposes. Generative Pre-trained Transformer 4 (GPT‑4, OpenAI, San Francisco, CA) has been evaluated for numerous clinical tasks and has been shown, among others, to be able to choose the correct imaging protocol in 84% of cases when provided with the radiology request form [15], or to present acceptable differential diagnoses in 94% of cases when provided with a description of imaging patterns [16]. Recently, GPT‑4 was shown to be able to correctly extract procedural data from free text reports in mechanical thrombectomy in acute ischemic stroke in 2631 out of 2800 (94.0%) of data points from 100 free text reports compared to the data entries of an experienced neuroradiologist, highlighting its potential to automize this labor-intensive and error-prone task [17]. While these results are promising, GPT‑4 can, to date, only be accessed via a browser version or via an Application Programming Interface (API), raising concerns on data safety when provided with medical data. Recently, the use of local large language models for the extraction of procedural data from thrombectomy reports was found to show high agreement with the data entries made by human readers [18]. Large Language Model Meta AI 3 (Llama 3, Meta, Menlo Park, CA) and Mixtral (Mistral AI, Paris, France) are large language models (LLM) that were released in April 2024 and in November 2023, respectively, and are both available for download and local use. Llama 3.1 405B was released in July 2024, and is also available for local use, given sufficient computing resources. The purpose of this study was to investigate whether Llama 3.1 405B, Llama 3 70B, Llama 3 8B, and Mixtral 8X7B can be used for data extraction from free text reports on mechanical thrombectomy with similar results as GPT‑4, and, thus represent data secure alternatives.

## Materials and Methods

### Ethics Approval

Institutional Review Board approval was obtained for this retrospective study and the need for written informed consent was waived.

### Study Sample

The study sample was the same as published previously [17]. In brief, consecutive reports from patients with ischemic stroke who underwent mechanical thrombectomy at institution 1 and another 30 reports from patients who underwent mechanical thrombectomy at institution 2 were extracted. De-identification was ensured by the neuroradiologist by manually deleting all personal information from the reports. Each report was given a pseudonym. The pseudonyms were not included in the data that was sent to the API of the respective LLM. Inclusion criteria were patient age > 18 years and intracranial artery occlusion applicable for mechanical thrombectomy. Exclusion criteria were absence of a detailed report or absence of intracranial vessel occlusion by digital subtraction angiography. No statistical analysis was performed to determine the study size.

### Prompt Generation

The prompt has been created in German Language and published previously [17] and was translated to English for this study. Both the prompt in German and English languages were formatted in JavaScript Object Notation (JSON). Each prompt was designed to be a single JSON element corresponding to individual requests, ensuring consistency and clarity in the prompt structure. Besides translating the German prompt to English language and formatting it in JSON format, the prompt remained unchanged compared to the previous publication. For each procedural detail, the LLMs were furnished with comprehensive instructions specifying the range of permissible data entries, such as “yes,” “no,” or “missing.” The 28 categories that were extracted from the reports, along with an explanation and the possible data entries provided to the LLMs for each procedural aspect, are summarized in Table 1. The full prompts in German and English language are provided as supplementary material S1 and S2.

**Table 1:** Categories extracted from the reports with an explanation and possible data entries

Category	Explanation
Date of Intervention	Date of the thrombectomy, format dd.mm.yyyy
Location of vessel occlusion	Location of the vessel occlusion. Possible entries: Carotid, Carotid terminus, M1, M2, M3, A1, A2, A3, basilar artery, P1, P2, P3, unknown
Side of vessel occlusion	Side of the vessel occlusion. Possible entries: left, right, not applicable
NIHSS	National Institutes of Health Stroke Scale. Missing, if not mentioned in the report
ASPECTS	Alberta Stroke Program Early CT Score. Missing, if not mentioned in the report
Intravenous Thrombolysis	Yes, if intravenous thromboloysis was administered. No, if it was not administered. Missing, if the report contains no information on intravenous thrombolysis
Symptom onset	Time of symptom onset, format hh:mm. Unknown, if the time of symptom onset was unknown. Missing, if the report mentions no time of symptom onset
Arrival at thrombectomy center	Time of the patient’s arrival at the thrombectomy center, format hh:mm. Missing, if the report mentions no time of arrival
Time of stroke imaging	Time of stroke imaging, format hh:mm. Missing, if the report mentions no time of stroke imaging
Groin puncture time	Time of groin puncture, format hh:mm. Missing, if the report mentions no time of groin puncture
Time of first intracranial run	Time of first intracranial run, format hh:mm. Missing, if the report mentions no time of first intracranial run
Time of first thrombectomy maneuver	Time of first thrombectomy maneuver, format hh:mm. Missing, if the report mentions no time of the first thrombectomy maneuver
Time of last thrombectomy maneuver	Time of last thrombectomy maneuver, format hh:mm. Missing, if the report mentions no time of the last thrombectomy maneuver. At our institution, the time of the last thrombectomy maneuver is used interchangably with the time of reperfusion
Final run	Time of the last intracranial run, format hh:mm. Missing, if the report mentions no time of the last intracranial run
Number of thrombectomy maneuvers	Number of thrombectomy maneuvers. Missing, if the report does not contain information on the number of thrombectomy maneuvers. 1, if the report mentions a first pass thrombectomy
mTICI	Final mTICI score. Possible entries: 0, 1, 2a, 2b, 2c, 3, missing, if no information on the recanalization score is included in the report. 3, if a complete reperfusion is mentioned in the report
BGC	Use of a balloon guide catheter. Possible entries: yes or no
Use of distal aspiration	Use of distal aspiration. Possible entries: yes or no
Use of stentretriever	Use of a stentretriever. Possible entries: yes or no
Extracranial stent implanted	Use of an extracranial stent. Possible entries: yes or no
Intracranial stent implanted	Use of an intracranial stent. Possible entries: yes or no
ASA	Administration of Acetylsalicylic Acid. Possible entries: yes or no
Clopidogrel	Administration of Clopidogrel. Possible entries: yes or no
Ticagrelor	Administration of Ticagrelor. Possible entries: yes or no
Tirofiban	Administration of Tirofiban. Possible entries: yes *or* no
Heparin	Administration of Heparin. Possible entries: yes or no
FDCT	Flat detector CT performed. Possible entries: yes or no
ICH	Intracranial hemorrhage detected. Possible entries: yes or no

*M1, M2, …* segments of the middle, anterior, or posterior cerebral artery, respectively, *NIHSS* National Institutes of Health Stroke Scale, *ASPECTS* Alberta Stroke Program Early CT Score, *mTICI* modified Thrombolysis in Cerebral Infarction, *BGC* balloon guide catheter, *ASA* Acetylsalicylic Acid, *FDCT* flat detector CT, *ICH* intracranial hemorrhage

### Experimental Setup

The experiments were conducted using the LLMs Llama 3.1 405B, Llama 3 70B, Llama 3 8B (all Meta AI), and Mixtral 8X7B (Mistral AI). For best comparability to the results of GPT published before [17], Llama 3.1 405B was provided with the prompt in German. The remaining models were evaluated using the English prompt, while the German prompt was additionally tested with the Llama 3 70B model to assess its multilingual capabilities. Except for Llama 3.1 405B, which was accessed via OpenRouter (https://openrouter.ai/), the LLMs were accessed and utilized via the Groq Application Programming Interface (API, https://groq.com/). For the Llama 3.1 405B and Llama 3 70B models, an optimized JSON-mode was employed, which enabled the model to generate responses directly in JSON format, ensuring a higher degree of accuracy and reducing the need for additional processing. In contrast, for the Mixtral 8X7B and Llama 3 8B models, JSON responses were requested, but due to the absence of an optimized JSON-mode, the responses often contained JSON data embedded within more extensive text outputs. In these cases, regular expressions were employed to parse and extract the relevant JSON data from the chat responses.

### Data Extraction from Thrombectomy Reports

The respective LLMs were provided with the free text reports alongside with the prompt. The responses from the LLMs were returned in JSON format, either directly or embedded within text. For Llama 3.1 405B and Llama 3 70B, the optimized JSON-mode provided a straightforward and accurate extraction of JSON data. For Mixtral 8X7B and Llama 8B, regular expressions were utilized to identify and extract the JSON data embedded within the broader textual responses. This method involved searching for JSON structures within the text and isolating them for further analysis. This dual approach to data extraction ensured that all responses were accurately captured and made suitable for subsequent analysis, maintaining the integrity and consistency of the data collected across different models.

### Evaluation of Data Entries by the LLMs and Statistical Analysis

The responses of the LLMs were collected as comma separated values (csv) tables and compared to the data entries of GPT‑4 as previously published [17] and the data entries of an interventional neuroradiologist with eight years of experience in diagnostic radiology and neuroradiology and three years of experience in interventional neuroradiology. Statistical analyses were conducted with R, version 4.4.1 (http://www.r-project.org/), and RStudio, version 2024.04.2 + 764 (https://posit.co/download/rstudio-desktop/). Also, precision, recall and F1 scores for each category were calculated. Interrater agreement was assessed by using Cohen’s kappa, which was assessed for Llama 3.1 405B, Llama 3 70B, Llama 3 8B, and Mixtral 8X7B against the neuroradiologist and GPT‑4. Additionally, Cohen’s kappa per category was calculated for each model against the neuroradiologist as reference standard. The strength of agreement for Kappa values is as follows: < 0.20 = poor, 0.21–0.40 = fair, 0.41–0.60 = moderate, 0.61–0.80 = good, and 0.81–1.00 = very good. For the comparison of Llama 3.1 405B, Llama 3 70B, Llama 3 8B, and Mixtral 8X7B versus the data entries of GPT‑4, McNemar’s test was used. The level of statistical significance was set at *P* = 0.05. No correction for multiple testing was performed due to the exploratory nature of this study.

## Results

### Study Sample

**Fig. 1:**
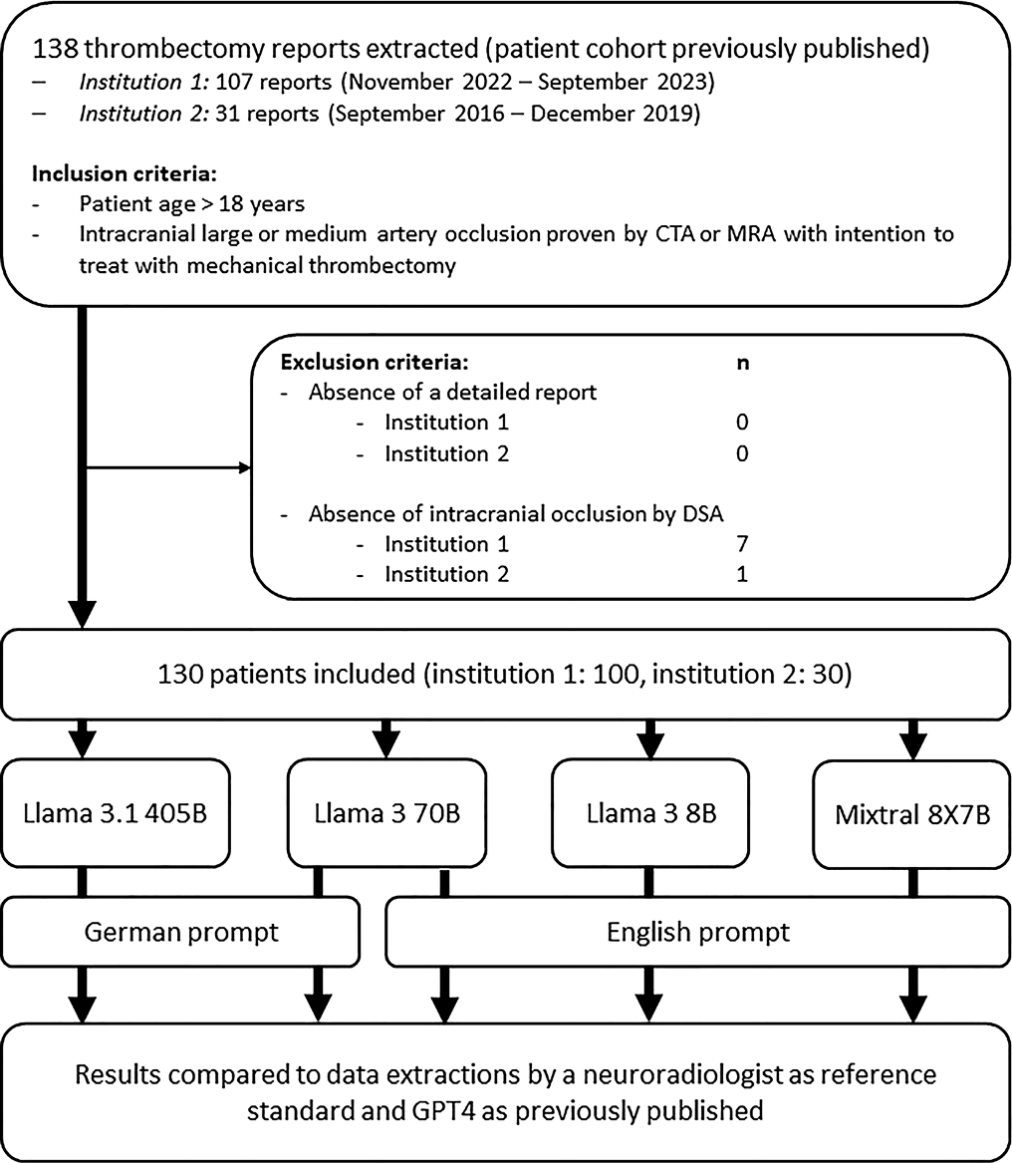
Flow Chart of the inclusion and exclusion process as well as the following data analysis. The patient cohort in this study has previously been published (*CTA* CT angiography, *MRA* MR angiography, *DSA* digital subtraction angiography)

The study sample was published before and remained unchanged for this study [17]. In brief, after exclusion of 7 patients due to absence of an intracranial occlusion, 100 reports on mechanical thrombectomy of 100 patients (mean age 74.7 ± 13.2 years; 47 males, 53 females), written by six different neurointerventionalists, were included in this study. Also, as published before, after exclusion of one report due to absence of an intracranial occlusion by DSA, 30 external reports from 30 patients of the second stroke center on mechanical thrombectomies performed between September 2016 and December 2019 (mean age 72.7 ± 13.5 years; 18 males, 12 females), written by five different neurointerventionalists were included in this study to evaluate the generalizability of the capabilities of the different LLMs. All reports were successfully processed by Llama 3.1 405B, Llama 3 70B, Llama 3 8B and Mixtral 8X7B. A flowchart of the inclusion and exclusion process as well as further data analysis is provided in Fig. 1. Patient characteristics and procedural details are summarized in Table 2.

**Table 2:** Patient characteristics

Characteristic	Institution 1	Institution 2
*Sex*		
Female	47 (47.0%)	12 (40.0%)
Male	53 (53.0%)	18 (60.0%)
*Mean age, years (± SD)*	74.7 ± 13.2	72.7 ± 13.5
*Age range, years*	23–98	39–92
*Occlusion site*		
ICA	19 (19.0%)	3 (10.0%)
M1	51 (51.0%)	19 (63.3%)
M2	22 (22.0%)	0 (0.0%)
A1	0 (0.0%)	0 (0.0%)
A2	0 (0.0%)	1 (3.3%)
BA	7 (7.0%)	7 (23.3%)
P1	0 (0.0%)	0 (0.0%)
P2	1 (1.0%)	0 (0.0%)
*Median NIHSS (range)*	8 (0–24)	Not applicable
*Median ASPECTS (range)*	9 (3–10)	Not applicable
*i.v. lysis*	23 (23.0%)	13 (43.3%)
*Door-to-groin time, minutes*	43 ± 24	Not applicable
*Groin-to-reperfusion time, minutes*	71 ± 172	28 ± 16
*mTICI*		
0	2 (2.0%)	1 (3.3%)
1	0 (0.0%)	0 (0.0%)
2a	3 (3.0%)	1 (3.3%)
2b	26 (26.0%)	15 (50.0%)
2c	20 (20.0%)	2 (6.7%)
3	48 (48.0%)	9 (30.0%)
*Mean number of maneuvers (± SD)*	2.9 ± 2.5	2.4 ± 1.9
*Extracranial stenting performed*	9 (9.0%)	2 (6.7%)
*Intracranial stenting performed*	2 (2.0%)	3 (10.0%)
*Postinterventional ICH*	17 (17.0%)	2 (6.7%)

Not applicable indicates that the information from the reports was insufficient to provide the respective information

The patient characteristics have previously been published and the table has been reprinted with permission [17]

*ICA* internal carotid artery, *M1, M2, A1, A2, P1, P2* segments of the middle, anterior and posterior cerebral arteries, respectively, *BA* basilar artery, *NIHSS* National Institutes of Health Stroke Scale score, *ASPECTS* Alberta Stroke Program Early CT Score, *mTICI* modified Thrombolysis in Cerebral Infarction, *ICH* intracranial hemorrhage

### Evaluation of Data Entries by the LLMs

A total of 2800 data entries was made by the neuroradiologist and by the LLMs, out of which Llama 3.1 405B, provided with the prompt in German, extracted 2619 (93.5% [95%CI: 92.6%, 94.4%]) data entries correctly, which was numerically lower than the 2631 (94.0%) data entries that were correctly extracted by GPT‑4, but without statistical significance (*p* = 0.39). Llama 3 70B, provided with the prompt translated to English, extracted 2537 correctly (90.6% [95% CI: 89.5%, 91.7%]), which was inferior to GPT‑4 (*p* < 0.001). Llama 3 70B provided with the prompt in German language extracted 2471 of the 2800 data entries (88.2% [95% CI: 87.0%, 89.4%]) correctly and thus was also inferior to GPT 4 (*p* < 0.001). Llama 3 8B provided with the English prompt achieved 2314 (82.6% [95% CI: 81.2%, 84.0%]) correct data entries and was also inferior to GPT‑4 (*p* < 0.001). Mixtral 8X7B achieved 2411 correct data entries (86.1% [95%CI: 84.8%, 87.4%]), also showing inferior results compared to GPT‑4 (*p* < 0.001). The rate of correct data entries ranged from 63.0% (95%CI: 52.8%, 72.4%) for the category *time of last thrombectomy maneuver* (which, at our institution, is used synonymously with ‘time of reperfusion’) to 100.0% (95% CI: 96.4%, 100.0%) for the categories *NIHSS, ASPECTS and use of stentretriever* for Llama 3.1 405B, from 42.0% (95%CI: 32.2%, 52.3%) for the category *intravenous thrombolysis* to 100.0% (95% CI: 96.4%, 100.0%) for the categories *ASPECTS and Tirofiban* for Llama 3 70b with the English prompt, from 44.0% (95% CI: 34.1%, 54.3%) for the category *intravenous thrombolysis* to 100.0% (95% CI: 96.4%, 100.0%) for the categories *ASPECTS and stentretriever* for Llama 3 70b with the German prompt, from 11.0% (95% CI: 5.6%, 18.3%) for the category *acetylsalicylic acid* to 100.0% (95% CI: 96.4%, 100.0%) for the categories *NIHSS and Tirofiban* for Llama 3 8b with the English prompt, and from 12.0% (95% CI: 6.4%, 20.0%) for the category *acetylsalicylic acid* to 100.0% (95% CI: 96.4%, 100.0%) for the categories *date of intervention, stentretriever *and *clopidogrel* for Mixtral 8X7B with the English prompt.

**Fig. 2:**
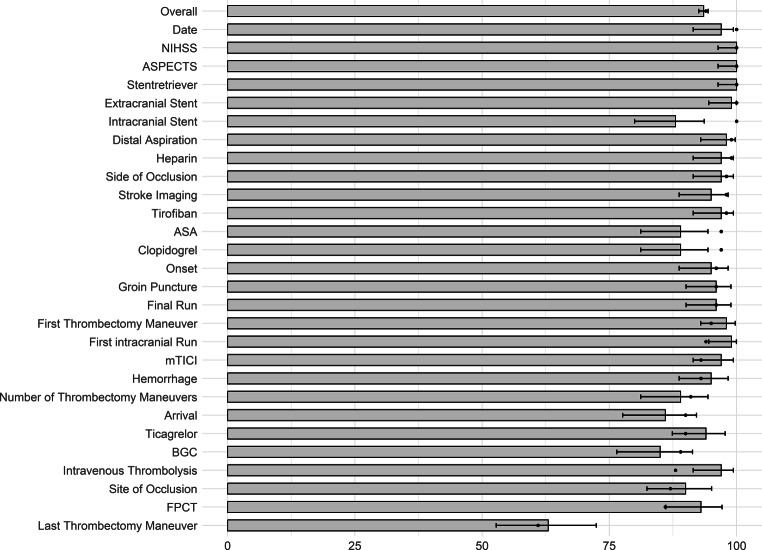
Rate of correct data entries by LlaMa3.1 405B. Grey bars represent the rate of correct data entries per category. *Errorbars *represent 95% confidence intervals. The *black dots* represent the rate of correct data entries made by GPT 4 as previously published (*NIHSS* National Institute of Health Stroke Scale, *ASPECTS* Alberta Stroke Program Early CT Score, *mTICI* modified Thrombolysis in Cerebral Infarction, *BGC* balloon guide catheter, *ASA* acetylsalicylic acid, *FPCT* flat panel computed tomography, *ICH* intracranial hemorrhage)

**Fig. 3:**
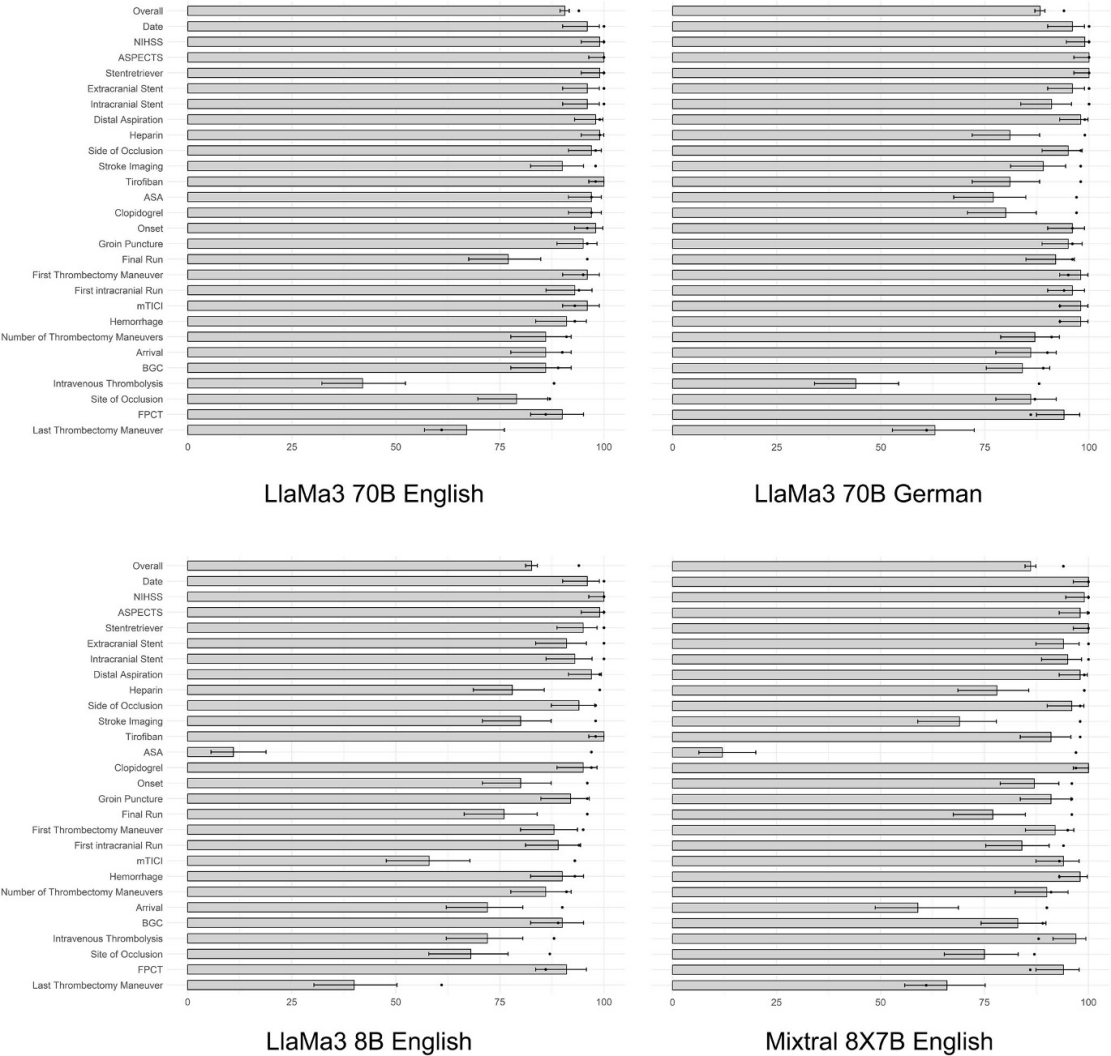
Rate of correct data entries by the large language models LlaMa3 70B, LlaMa3 8B, and Mixtral 8X7B. *Grey bars* represent the rate of correct data entries per category. *Errorbars *represent 95% confidence intervals for the respective large language model. The *black dots* represent the rate of correct data entries made by GPT 4 as previously published (*NIHSS* National Institute of Health Stroke Scale, *ASPECTS* Alberta Stroke Program Early CT Score, *mTICI* modified Thrombolysis in Cerebral Infarction, *BGC* balloon guide catheter, *ASA* acetylsalicylic acid, *FPCT* flat panel computed tomography, *ICH* intracranial hemorrhage)

For the 30 reports from center 2, 840 data entries were made by the neuroradiologist and by the LLMs, out of which Llama 3.1 405B with the German Prompt extracted 774 correctly (92.1% [95% CI: 90.1%, 93.9%]), which was numerically higher than the 760 (90.5%) data entries that were correctly extracted by GPT‑4 (*p* = 0.63). Llama 3 70B, with the English prompt, extracted 722 correctly (86.0% [95% CI: 83.4%, 88.2%]), which was lower than the rate of correct data entries by GPT‑4 (*p* < 0.001). Llama 3 70B provided with the German prompt extracted 743 of the 840 data entries (88.5% [95% CI: 86.1%, 90.5%]) correctly, also inferior to GPT 4 (*p* < 0.001). Llama 3 8B with the English prompt achieved 586 (69.8% [95% CI: 66.5%, 72.9%]) correct data entries and was also inferior to GPT‑4 (*p* < 0.001). Mixtral 8X7B achieved 652 correct data entries (77.6% [95%CI: 74.6%, 80.4%]), also showing inferior results compared to GPT‑4 (*p* < 0.001). The rate of correct data entries ranged from 46.7% (95%CI: 28.3%, 65.7%) for the category *time of groin puncture *to 100.0% (95% CI: 88.4%, 100.0%) for the categories *date of intervention, NIHSS, ASPECTS, symptom onset, arrival at thrombectomy center, time of stroke imaging, time of first intracranial run, time of first thrombectomy maneuver, use of stentretriever, extracranial stent, ticagrelor, tirofiban, heparin, and hemorrhage*, from 36.7% (95%CI: 19.9%, 56.1%) for the category *last thrombectomy maneuver *to 100.0% (95% CI: 88.4%, 100.0%) for the categories *date of intervention, NIHSS, ASPECTS, time of arrival at thrombectomy center, stentretriever, extracranial stent, intracranial stent, Tirofiban, Heparin, and Hemorrhage* for Llama 3 70b with the English prompt, from 30.0% (95% CI: 14.7%, 49.4%) for the category *number of thrombectomy maneuvers* to 100.0% (95% CI: 88.4%, 100.0%) for the categories *date of intervention, NIHSS, ASPECTS, arrival at thrombectomy center, time of stroke imaging, stentretriever, extracranial stent, Tirofiban, Heparin, and Hemorrhage* for Llama 3 70b with the German prompt, from 16.7% (95% CI: 5.6%, 34.7%) for the category *acetylsalicylic acid* to 100.0% (95% CI: 88.4%, 100.0%) for the categories *date of intervention, NIHSS, ASPECTS, extracranial stent, Tirofiban, Heparin, and hemorrhage *for Llama 3 8b with the English prompt, and from 16.7% (95% CI: 5.6%, 34.7%) for the category *acetylsalicylic acid* to 100.0% (95% CI: 88.4%, 100.0%) for the categories *date of intervention, NIHSS, ASPECTS, extracranial stent, heparin, intracranial stent and stentretriever* for Mixtral 8X7B with the English prompt. Results of the rate of correct data entries are visualized in Fig. 2 for the internal reports for Llama 3.1 405B, and in Fig. 3 for the internal reports for the remaining LLMs, and results for all LLMs are summarized in Table 3 (center 1) and Table 4 (center 2). Precision, recall and F1-scores are summarized in supplementary tables S3 (center 1) and S4 (center 2).

**Table 3:** Rate of correct data entries made by the respective large language models for the 100 internal reports

Category	Correct data entries GPT‑4 (%)	Correct data entries Llama 3.1 405B German prompt (%)	*p* GPT‑4 vs Llama 3.1 405B German prompt	Correct data entries Llama 3 70b English prompt (%)	*p* GPT‑4 vs Llama 3 70b English prompt	Correct data entries Llama 3 70b German prompt (%)	*p* GPT‑4 vs Llama 3 70b German prompt	Correct data entries Llama 3 8b English prompt (%)	*p* GPT‑4 vs Llama 3 8b English prompt	Correct data entries Mixtral 8X7B English prompt (%)	*p* GPT‑4 vs Mixtral 8X7B English prompt
Overall	94.0 (93.0, 94.8)	93.5 (92.6, 94.4)	0.385	90.6 (89.5, 91.7)	< 0.001	88.2 (87.0, 89.4)	< 0.001	82.6 (81.2, 84.0)	< 0.001	86.1 (84.8, 87.4)	< 0.001
Date of Intervention	100.0 (96.4, 100.0)	97.0 (91.5, 99.4)	0.248	96.0 (90.1, 98.9)	0.134	96.0 (90.1, 98.9)	0.134	96.0 (90.1, 98.9)	0.134	100.0 (96.4, 100.0)	NaN
Location of vessel occlusion	87.0 (78.8, 92.9)	90.0 (82.4, 95.1)	0.371	79.0 (69.7, 86.5)	0.08	86.0 (77.6, 92.1)	1	68.0 (57.9, 77.0)	0.002	75.0 (65.3, 83.1)	0.006
Side of vessel occlusion	98.0 (93.0, 99.8)	97.0 (91.5, 99.4)	1	97.0 (91.5, 99.4)	1	95.0 (88.7, 98.4)	0.371	94.0 (87.4, 97.8)	0.221	96.0 (90.1, 98.9)	0.617
NIHSS	100.0 (96.4, 100.0)	100.0 (96.4, 100.0)	NaN	99.0 (94.6, 100.0)	1	99.0 (94.6, 100.0)	1	100.0 (96.4, 100.0)	NaN	99.0 (94.6, 100.0)	1
ASPECTS	100.0 (96.4, 100.0)	100.0 (96.4, 100.0)	NaN	100.0 (96.4, 100.0)	NaN	100.0 (96.4, 100.0)	NaN	99.0 (94.6, 100.0)	1	98.0 (93.0, 99.8)	0.48
Intravenous Thrombolysis	88.0 (80.0, 93.6)	97.0 (91.5, 99.4)	0.016	42.0 (32.2, 52.3)	< 0.001	44.0 (34.1, 54.3)	< 0.001	72.0 (62.1, 80.5)	0.015	97.0 (91.5, 99.4)	0.039
Symptom onset	96.0 (90.1, 98.9)	95.0 (88.7, 98.4)	1	98.0 (93.0, 99.8)	0.48	96.0 (90.1, 98.9)	1	80.0 (70.8, 87.3)	0.001	87.0 (78.8, 92.9)	0.039
Arrival at thrombectomy center	90.0 (82.4, 95.1)	86.0 (77.6, 92.1)	0.221	86.0 (77.6, 92.1)	0.289	86.0 (77.6, 92.1)	0.221	72.0 (62.1, 80.5)	< 0.001	59.0 (48.7, 68.7)	< 0.001
Time of stroke imaging	98.0 (93.0, 99.8)	95.0 (88.7, 98.4)	0.45	90.0 (82.4, 95.1)	0.043	89.0 (81.2, 94.4)	0.027	80.0 (70.8, 87.3)	< 0.001	69.0 (59.0, 77.9)	< 0.001
Groin puncture time	96.0 (90.1, 98.9)	96.0 (90.1, 98.9)	NaN	95.0 (88.7, 98.4)	1	95.0 (88.7, 98.4)	1	92.0 (84.8, 96.5)	0.134	91.0 (83.6, 95.8)	0.131
Time of first intracranial run	94.0 (87.4, 97.8)	99.0 (94.6, 100.0)	0.131	93.0 (86.1, 97.1)	1	96.0 (90.1, 98.9)	0.683	89.0 (81.2, 94.4)	0.302	84.0 (75.3, 90.6)	0.016
Time of first thrombectomy maneuver	95.0 (88.7, 98.4)	98.0 (93.0, 99.8)	0.45	96.0 (90.1, 98.9)	1	98.0 (93.0, 99.8)	0.45	88.0 (80.0, 93.6)	0.096	92.0 (84.8, 96.5)	0.505
Time of last thrombectomy maneuver	61.0 (50.7, 70.6)	63.0 (52.8, 72.4)	0.752	67.0 (56.9, 76.1)	0.149	63.0 (52.8, 72.4)	0.823	40.0 (30.3, 50.3)	< 0.001	66.0 (55.8, 75.2)	0.359
Final run	96.0 (90.1, 98.9)	96.0 (90.1, 98.9)	1	77.0 (67.5, 84.8)	0	92.0 (84.8, 96.5)	0.221	76.0 (66.4, 84.0)	< 0.001	77.0 (67.5, 84.8)	< 0.001
Number of thrombectomy maneuvers	91.0 (83.6, 95.8)	89.0 (81.2, 94.4)	0.617	86.0 (77.6, 92.1)	0.131	87.0 (78.8, 92.9)	0.221	86.0 (77.6, 92.1)	0.182	90.0 (82.4, 95.1)	1
mTICI	93.0 (86.1, 97.1)	97.0 (91.5, 99.4)	0.221	96.0 (90.1, 98.9)	0.45	98.0 (93.0, 99.8)	0.131	58.0 (47.7, 67.8)	< 0.001	94.0 (87.4, 97.8)	1
BGC	89.0 (81.2, 94.4)	85.0 (76.5, 91.4)	0.134	86.0 (77.6, 92.1)	0.248	84.0 (75.3, 90.6)	0.074	90.0 (82.4, 95.1)	1	83.0 (74.2, 89.8)	0.239
Use of distal aspiration	99.0 (94.6, 100.0)	98.0 (93.0, 99.8)	1	98.0 (93.0, 99.8)	1	98.0 (93.0, 99.8)	1	97.0 (91.5, 99.4)	0.617	98.0 (93.0, 99.8)	1
Use of stentretriever	100.0 (96.4, 100.0)	100.0 (96.4, 100.0)	NaN	99.0 (94.6, 100.0)	1	100.0 (96.4, 100.0)	NaN	95.0 (88.7, 98.4)	0.074	100.0 (96.4, 100.0)	NaN
Extracranial stent implanted	100.0 (96.4, 100.0)	99.0 (94.6, 100.0)	1	96.0 (90.1, 98.9)	0.134	96.0 (90.1, 98.9)	0.134	91.0 (83.6, 95.8)	0.008	94.0 (87.4, 97.8)	0.041
Intracranial stent implanted	100.0 (96.4, 100.0)	88.0 (80.0, 93.6)	0.001	96.0 (90.1, 98.9)	0.134	91.0 (83.6, 95.8)	0.008	93.0 (86.1, 97.1)	0.023	95.0 (88.7, 98.4)	0.074
ASA	97.0 (91.5, 99.4)	89.0 (81.2, 94.4)	0.061	97.0 (91.5, 99.4)	1	77.0 (67.5, 84.8)	< 0.001	11.0 (5.6, 18.8)	< 0.001	12.0 (6.4, 20.0)	< 0.001
Clopidogrel	97.0 (91.5, 99.4)	89.0 (81.2, 94.4)	0.061	97.0 (91.5, 99.4)	1	80.0 (70.8, 87.3)	< 0.001	95.0 (88.7, 98.4)	0.683	100.0 (96.4, 100.0)	0.248
Ticagrelor	90.0 (82.4, 95.1)	94.0 (87.4, 97.8)	0.343	91.0 (83.6, 95.8)	1	71.0 (61.1, 79.6)	< 0.001	93.0 (86.1, 97.1)	0.45	94.0 (87.4, 97.8)	0.289
Tirofiban	98.0 (93.0, 99.8)	97.0 (91.5, 99.4)	1	100.0 (96.4, 100.0)	0.48	81.0 (71.9, 88.2)	< 0.001	100.0 (96.4, 100.0)	0.48	91.0 (83.6, 95.8)	0.046
Heparin	99.0 (94.6, 100.0)	97.0 (91.5, 99.4)	0.48	99.0 (94.6, 100.0)	NaN	81.0 (71.9, 88.2)	< 0.001	78.0 (68.6, 85.7)	< 0.001	78.0 (68.6, 85.7)	< 0.001
FDCT	86.0 (77.6, 92.1)	93.0 (86.1, 97.1)	0.096	90.0 (82.4, 95.1)	0.423	94.0 (87.4, 97.8)	0.027	91.0 (83.6, 95.8)	0.302	94.0 (87.4, 97.8)	0.043
ICH	93.0 (86.1, 97.1)	95.0 (88.7, 98.4)	0.773	91.0 (83.6, 95.8)	0.803	98.0 (93.0, 99.8)	0.182	90.0 (82.4, 95.1)	0.628	98.0 (93.0, 99.8)	0.182

95% confidence intervals are given in parentheses

The results of GPT‑4 have been previously published and were not re-evaluated

Statistical significance was determined with McNemar’s test

*NIHSS* National Institutes of Health Stroke Scale, *ASPECTS* Alberta Stroke Program Early CT Score, *mTICI* modified Thrombolysis in Cerebral Infarction, *BGC* balloon guide catheter, *ASA* acetylsalicylic acid, *FPCT* flat panel computed tomography, *ICH* intracranial hemorrhage, *NaN* Not a Number (McNemar test not possible due to division by 0)

**Table 4:** Rate of correct data entries made by the respective large language models for the 30 external reports

Category	Correct data entries GPT‑4 (%)	Correct data entries Llama 3.1 405B German prompt (%)	*p* GPT‑4 vs Llama 3.1 405B German prompt	Correct data entries Llama 3 70b English prompt (%)	*p* GPT‑4 vs Llama 3 70b English prompt	Correct data entries Llama 3 70b German prompt (%)	*p* GPT‑4 vs Llama 3 70b German prompt	Correct data entries Llama 3 8b English prompt (%)	*p* GPT‑4 vs Llama 3 8b English prompt	Correct data entries Mixtral 8X7B English prompt (%)	*p* GPT‑4 vs Mixtral 8X7B English prompt
Overall	90.5 (88.3, 92.4)	92.1 (90.1, 93.9)	0.626	86.0 (83.4, 88.2)	< 0.001	88.5 (86.1, 90.5)	< 0.001	69.8 (66.5, 72.9)	< 0.001	77.6 (74.6, 80.4)	< 0.001
Date of Intervention	100.0 (88.4, 100.0)	100.0 (88.4, 100.0)	NaN	100.0 (88.4, 100.0)	NaN	100.0 (88.4, 100.0)	NaN	100.0 (88.4, 100.0)	NaN	100.0 (88.4, 100.0)	NaN
Location of vessel occlusion	90.0 (73.5, 97.9)	86.7 (69.3, 96.2)	1	93.3 (77.9, 99.2)	1	90.0 (73.5, 97.9)	1	53.3 (34.3, 71.7)	0.01	73.3 (54.1, 87.7)	0.131
Side of vessel occlusion	93.3 (77.9, 99.2)	96.7 (82.8, 99.9)	1	83.3 (65.3, 94.4)	0.37	80.0 (61.4, 92.3)	0.221	73.3 (54.1, 87.7)	0.077	96.7 (82.8, 99.9)	1
NIHSS	100.0 (88.4, 100.0)	100.0 (88.4, 100.0)	NaN	100.0 (88.4, 100.0)	NaN	100.0 (88.4, 100.0)	NaN	100.0 (88.4, 100.0)	NaN	100.0 (88.4, 100.0)	NaN
ASPECTS	100.0 (88.4, 100.0)	100.0 (88.4, 100.0)	NaN	100.0 (88.4, 100.0)	NaN	100.0 (88.4, 100.0)	NaN	100.0 (88.4, 100.0)	NaN	100.0 (88.4, 100.0)	NaN
Intravenous Thrombolysis	93.3 (77.9, 99.2)	90.0 (73.5, 97.9)	1	60.0 (40.6, 77.3)	0.004	76.7 (57.7, 90.1)	0.074	33.3 (17.3, 52.8)	< 0.001	70.0 (50.6, 85.3)	0.023
Symptom onset	83.3 (65.3, 94.4)	100.0 (88.4, 100.0)	1	76.7 (57.7, 90.1)	0.041	93.3 (77.9, 99.2)	1	53.3 (34.3, 71.7)	0.001	53.3 (34.3, 71.7)	0.001
Arrival at thrombectomy center	100.0 (88.4, 100.0)	100.0 (88.4, 100.0)	NaN	100.0 (88.4, 100.0)	NaN	100.0 (88.4, 100.0)	NaN	20.0 (7.7, 38.6)	< 0.001	40.0 (22.7, 59.4)	< 0.001
Time of stroke imaging	86.7 (69.3, 96.2)	100.0 (88.4, 100.0)	0.48	93.3 (77.9, 99.2)	1	100.0 (88.4, 100.0)	0.48	53.3 (34.3, 71.7)	0.001	36.7 (19.9, 56.1)	< 0.001
Groin puncture time	56.7 (37.4, 74.5)	46.7 (28.3, 65.7)	0.221	43.3 (25.5, 62.6)	0.074	43.3 (25.5, 62.6)	0.074	46.7 (28.3, 65.7)	0.221	50.0 (31.3, 68.7)	0.579
Time of first intracranial run	96.7 (82.8, 99.9)	100.0 (88.4, 100.0)	NaN	96.7 (82.8, 99.9)	1	90.0 (73.5, 97.9)	0.248	40.0 (22.7, 59.4)	< 0.001	73.3 (54.1, 87.7)	0.013
Time of first thrombectomy maneuver	90.0 (73.5, 97.9)	100.0 (88.4, 100.0)	0.48	66.7 (47.2, 82.7)	0.027	86.7 (69.3, 96.2)	0.48	43.3 (25.5, 62.6)	< 0.001	43.3 (25.5, 62.6)	< 0.001
Time of last thrombectomy maneuver	53.3 (34.3, 71.2)	63.3 (43.9, 80.1)	0.248	36.7 (19.9, 56.1)	0.228	70.0 (50.6, 85.3)	0.131	33.3 (17.3, 52.8)	0.077	46.7 (28.3, 65.7)	0.617
Final run	93.3 (77.9, 99.2)	96.7 (82.8, 99.9)	1	76.7 (57.7, 90.1)	0.041	93.3 (77.9, 99.2)	1	73.3 (54.1, 87.7)	0.023	86.7 (69.3, 96.2)	0.371
Number of thrombectomy maneuvers	46.7 (28.3, 65.7)	60.0 (40.6, 77.3)	0.221	50.0 (31.3, 68.7)	1	30.0 (14.7, 49.4)	0.131	46.7 (28.3, 65.7)	1	60.0 (40.6, 77.3)	0.343
mTICI	93.3 (77.9, 99.2)	96.7 (82.8, 99.9)	1	93.3 (77.9, 99.2)	NaN	93.3 (77.9, 99.2)	NaN	53.3 (34.3, 71.7)	0.003	90.0 (73.5, 97.9)	1
BGC	90.0 (73.5, 97.9)	90.0 (73.5, 97.9)	NaN	90.0 (73.5, 97.9)	NaN	90.0 (73.5, 97.9)	NaN	90.0 (73.5, 97.9)	NaN	90.0 (73.5, 97.9)	NaN
Use of distal aspiration	100.0 (88.4, 100.0)	96.7 (82.8, 99.9)	1	96.7 (82.8, 99.9)	1	96.7 (82.8, 99.9)	1	96.7 (82.8, 99.9)	1	96.7 (82.8, 99.9)	1
Use of stentretriever	93.3 (77.9, 99.2)	100.0 (88.4, 100.0)	0.48	100.0 (88.4, 100.0)	0.48	100.0 (88.4, 100.0)	0.48	63.3 (43.9, 80.1)	0.027	100.0 (88.4, 100.0)	0.48
Extracranial stent implanted	100.0 (88.4, 100.0)	100.0 (88.4, 100.0)	NaN	100.0 (88.4, 100.0)	NaN	100.0 (88.4, 100.0)	NaN	100.0 (88.4, 100.0)	NaN	100.0 (88.4, 100.0)	NaN
Intracranial stent implanted	100.0 (88.4, 100.0)	96.7 (82.8, 99.9)	1	100.0 (88.4, 100.0)	NaN	96.7 (82.8, 99.9)	1	96.7 (82.8, 99.9)	1	100.0 (88.4, 100.0)	NaN
ASA	96.7 (82.8, 99.9)	96.7 (82.8, 99.9)	1	83.3 (65.3, 94.4)	0.134	80.0 (61.4, 92.3)	0.074	16.7 (5.6, 34.7)	< 0.001	16.7 (5.6, 34.7)	< 0.001
Clopidogrel	93.3 (77.9, 99.2)	86.7 (69.3, 96.2)	0.48	90.0 (73.5, 97.9)	1	90.0 (73.5, 97.9)	1	90.0 (73.5, 97.9)	1	93.3 (77.9, 99.2)	1
Ticagrelor	100.0 (88.4, 100.0)	100.0 (88.4, 100.0)	NaN	100.0 (88.4, 100.0)	NaN	100.0 (88.4, 100.0)	NaN	100.0 (88.4, 100.0)	NaN	100.0 (88.4, 100.0)	NaN
Tirofiban	100.0 (88.4, 100.0)	100.0 (88.4, 100.0)	NaN	100.0 (88.4, 100.0)	NaN	100.0 (88.4, 100.0)	NaN	100.0 (88.4, 100.0)	NaN	83.3 (65.3, 94.4)	0.074
Heparin	100.0 (88.4, 100.0)	100.0 (88.4, 100.0)	NaN	100.0 (88.4, 100.0)	NaN	100.0 (88.4, 100.0)	NaN	100.0 (88.4, 100.0)	NaN	100.0 (88.4, 100.0)	NaN
FDCT	83.3 (65.3, 94.4)	76.7 (57.7, 90.1)	0.48	76.7 (57.7, 90.1)	0.48	76.7 (57.7, 90.1)	0.48	76.7 (57.7, 90.1)	0.48	76.7 (57.7, 90.1)	0.48
ICH	100.0 (88.4, 100.0)	100.0 (88.4, 100.0)	NaN	100.0 (88.4, 100.0)	NaN	100.0 (88.4, 100.0)	NaN	100.0 (88.4, 100.0)	NaN	96.7 (82.8, 99.9)	1

95% confidence intervals are given in parentheses

The results of GPT‑4 have been previously published and were not re-evaluated

Statistical significance was determined with McNemar’s test

*NIHSS* National Institutes of Health Stroke Scale, *ASPECTS* Alberta Stroke Program Early CT Score, *mTICI* modified Thrombolysis in Cerebral Infarction, *BGC* balloon guide catheter, *ASA* acetylsalicylic acid, *FPCT* flat panel computed tomography, *ICH* intracranial hemorrhage, *NaN* Not a Number (McNemar test not possible due to division by 0)

No difference was found for the internal reports when Llama 3 70B was provided with the German or the English prompt (*p* = 0.71), but for the external reports, Llama 3 70B, provided with the prompt in German, was superior to Llama 3 70B, when provided with the prompt in English (*p* = 0.005).

Cohen’s kappa for the internal reports ranged from 0.81 (Llama 3 8B, very good) to 0.93 (Llama 3.1 405B with German prompt, very good) against GPT‑4, and from 0.80 (Llama 3 8B, good) to 0.93 (Llama 3.1 405B with German prompt, very good) against the neuroradiologist. Cohen’s kappa for the external reports ranged from 0.69 (Llama 3 8B, good) to 0.94 (Llama 3 405B with German prompt, very good) against GPT‑4 and from 0.64 (Llama 3 8B, good) to 0.90 (Llama 3 405B with German prompt, very good) against the neuroradiologist. Per category, Cohen’s kappa ranged from 0.00 (for example, for the categories Clopidogrel and Ticagrelor for Llama 3.1 405B) to 1.00 (for example, for the categorie use of stentretriever for Llama 3.1 405B). Overall Kappa statistics are summarized in Table 5. Kappa values per model and category are summarized in supplementary table S5.

**Table 5:** Cohen’s kappa of the four large language models against GPT‑4 and the neuroradiologist

Model	GPT‑4	*p*	Neuroradiologist	*p*
*Internal reports*				
Llama 3.1 405B German prompt	0.93	< 0.001	0.93	< 0.001
Llama 3 70B English prompt	0.9	< 0.001	0.89	< 0.001
Llama 3 70B German prompt	0.88	< 0.001	0.87	< 0.001
Llama 3 8B English prompt	0.81	< 0.001	0.8	< 0.001
Mixtral 8X7B English prompt	0.84	< 0.001	0.84	< 0.001
*External reports*				
Llama 3.1 405B German prompt	0.94	< 0.001	0.9	< 0.001
Llama 3 70B English prompt	0.87	< 0.001	0.83	< 0.001
Llama 3 70B German prompt	0.91	< 0.001	0.86	< 0.001
Llama 3 8B English prompt	0.69	< 0.001	0.64	< 0.001
Mixtral 8X7B English prompt	0.76	< 0.001	0.73	< 0.001

## Discussion

GPT‑4 has been shown to correctly extract procedural data from free text reports on thrombectomy procedures in acute ischemic stroke in 94.0% of data entries, showing the potential of state of the art large language models to assist human readers in collecting data for clinical studies or stroke registries [17]. While GPT‑4 can, to date, only be accessed either via a browser interface or an API with the need to anonymize data prior to analysis to avoid violation of data protection laws. Thus, in this retrospective study, we evaluated the ability of Llama 3.1 405B, the largest and most recent LLM by Meta AI, as well as Llama 3 70B and 8B, and Mixtral8X7B, which are recent LLMs that can all be installed and run on local servers, to extract data from free text reports on mechanical thrombectomies in acute ischemic stroke. Our current study was similar to the study previously published [17], thus, our current results are highly comparable to those published before. Llama 3.1 405B with the same German prompt that was previously used for GPT‑4 [17], extracted 93.5% of data entries correctly, which was comparable to the results shown by GPT‑4 as previously published [17]. Llama 3 70B with the prompt either in German or English with 90.4% and 88.3% correct data entries, respectively, was inferior to GPT 4 and Llama 3.1 405B, although still showing high agreement with the reference standard. Both Mixtral 8X7B with 86.1% correct data entries and Llama 3 8B with 82.6% correct data entries were inferior to GPT‑4 (both provided with the prompt translated to English). In the external dataset, Llama 3.1 405B with the German prompt achieved 92.1%, Llama 3 70B achieved 86.0% (English prompt)and 88.5% (German prompt), Llama 3 8B achieved69.8% (English prompt), and Mixtral 8X7B achieved 77.6% correct data entries.

While numerous studies have been published on the abilities of GPT 4 for medical purposes, fewer studies have evaluated less known alternatives. Recently, Meddeb et al. [18] published their results on data extraction from unstructured thrombectomy reports using the local and open source large language models Mixtral, Qwen, and BioMistral for 15 categories, and found high agreement between the output of the models and the reference standard, with Mixtral showing the best performance, with precision values ranging from 68.0–99.0% for the different categories, which is mostly in line with our results.

In the category ‘ASA’, Llama 3 8B and Mixtral 8X7B produced strikingly poor results, with only 11% or 12% correct data entries, respectively. Both models entered ‘yes’ in this category in > 90% of cases, although, according to the reference standard, ASA was only administered in 9% of the procedures. The other models used in this study achieved > 70% correct data entries in this category. Also, for the other categories that included medication administered during the procedures, Llama 3 8B and Mixtral 8X7B achieved more acceptable results that were comparable to the other models. While we do not have a definitive explanation for these discrepancies, we believe that the different number of parameters utilized by the models determines their ability to interpret text accurately. Thus, models using more parameters like Llama 3.1 405B and Llama 3 70B may have been more capable to interpret the reports and the prompt, and thus to give correct date entries, while smaller models, like Llama 3 8B, may have entered generic answers, more relying on the probability of a drug being administered than actually interpreting the reports. This may explain the high frequency of ‘yes’ for the category ‘ASA’, representing a drug being used in many different situations, as opposed to other medication like Clopidogrel or, even more, Tirofiban, being reserved for more specific situations and thus, probably, being underrepresented in the training data of the models.

While GPT‑4 is easy to use and outperformed most of the LLMs evaluated in this study except for Llama 3.1 405B, there may be concerns regarding privacy and data security when using it for medical data, requiring elaborate anonymization of reports prior to data processing to avoid re-identificiation [19]. Llama 3.1 405B was comparable to GPT for the specific task of data extraction from reports on mechanical thrombectomy, but, when run locally, is in need for extensive computing resources. Llama 3 70B, Llama 3 8B and Mixtral8X7B were outperformed by GPT‑4 for the task of data extraction from thrombectomy reports in our study, however, they can, like Llama 3.1 405B, be run locally and offline, making it possible to utilize them for similar tasks without the need for anonymization. Although inferior to GPT‑4, we regard the rate of correct data entries especially made by Llama 3 70B as promising, especially because the prompt that was optimized for GPT‑4 and remained unchanged for this study. We believe that Llama 3 70B may represent a reasonable compromise and further improvements are possible in future studies, evaluating the more recent versions of Llama 3.1 70B. While Llama 3 8B and Mixtral 8X7B showed promising results on the 100 internal reports, they both showed a greater decrease of performance when used for the external reports and thus, apparently, a lesser generalizability. Regulatory aspects, especially in the European Union, may limit the use of Llama 3 and its future versions in the future.

We found no significant differences between the results entered by Llama 3 70B when provided with the prompt in English language or in German for the 100 internal reports (*p* = 0.71). In contrast, for the 30 external reports, we found Llama 3 70B to be significantly superior when provided with the prompt in German language instead of English (*p* = 0.005). These equivocal results may motivate further research in the future to determine the model’s multilingual capabilities. In general, there have been multiple studies that assessed multillingual capacities of different LLMs in the past. They found that GPT 3.5 performed best in English language compared to Spanish, Chinese, and Hindi, respectively, when tested on questions concerning the healthcare system [20]. Ahuja et al. [21] reported that, when tested for their multilinguality, state of the art LLMs performed significantly better in Germanic languages than in other language families, like Bantu and Afro-Asiatic. Given the fact, that specific research has been dealing with differences in performances of LLMs in different languages, and that both German and English belong to the Germanic language family, the additional information of our results is limited.

We are aware of several limitations of our study. First, its retrospective nature may limit its generalizability to other institutions. Second, it has been shown that prompt optimization may not only be dependent on the specific task, but also on the model that is used [22]. The prompt that was previously [17] used for GPT‑4 was not optimized for Llama 3/Llama 3.1 or Mixtral, thus, the performance of the latter models may be underestimated for the purpose of better comparability. Third, since GPT‑4 was evaluated for the purpose of data extraction from thrombectomy reports, it has been repeatedly updated, thus, the results that were previously published may already be outdated. This issue may be overcome when an LLM can be run in a local, offline environment, increasing control over software updates and changes of the model’s capabilities. Fourth, although intended for local installation and use, this study was not executed on a local LLM, but on the respective API with anonymized reports. Thus, our results may need confirmation as soon as a local LLM has been established. We chose to perform our study with remote servers to ensure our ability to evaluate very large models beyond the smaller 8B versions. The Llama 3.1 405B model comes with around 2.3 TB (Terabytes) worth of parameters, evaluation on this scale is beyond the means of our institution. However, for feasibility purposes, we have set up a local version of Llama 3.1 8B at our institution and would like to share our experience. On disk the 8B instruct model consumes about 13 Gigabytes of space and runs comfortably on a Nvidia A40 GPU, consuming about 18 GB of GPU-Ram. The process of setting up the local version of Llama 3.1 8B was as follows: First, an account was established on a server available in a local network equipped with an Nvidia A40 GPU or equivalent. Then, the license from the LLM provider was accepted. With this license, a Huggingface access key was obtained. Subsequently, Python was set up for local inference, and dependencies like PyTorch were installed using a package manager. Finally, the reports were looped over and the prompt was run.

In conclusion, we found that Llama 3.1 405B was comparable to GPT‑4, and Llama 3 70B, Llama 3 8B, and Mixtral 8X7B were outperformed by GPT‑4 for the purpose of data extraction from reports on mechanical thrombectomy in acute ischemic stroke. Thus, Llama 3.1 405B, given sufficient computing capacities, can be regarded as an alternative to GPT‑4. Also, Llama 3 70B showed promising results and may represent both a reasonable alternative to GPT‑4 and Llama 3.1 405B in terms of performance and computing capacities when run in a local environment.

## Supplementary Information

**Supplementary material S1. **Prompt given to the LLMs in English language

**Supplementary material S2. **Prompt given to the LLMs in English language

**Supplementary table S3. **Precision, recall, and F1 scores for the internal reports from center 1

**Supplementary table S4. **Precision, recall, and F1 scores for the external reports from center 2

**Table S5. **Cohen’s kappa of the different models against the neuroradiologist by category

## Data Availability

Data will be made available upon reasonable request by the corresponding author.
