# Supplementary material for: Llama 3.1 405B Is Comparable to GPT-4 for Extraction of Data from Thrombectomy Reports—A Step Towards Secure Data Extraction
Source: Clin Neuroradiol. 2025 Feb 25;35(3):495–510. doi: 10.1007/s00062-025-01500-z (PMC12454497; doi:10.1007/s00062-025-01500-z)
Supplement: Supplementary file 3 — Supplementary table S3. Precision, recall, and F1 scores for the internal reports from center 1 [file 62_2025_1500_MOESM3_ESM.docx]

**Supplementary table S3.** Precision, recall, and F1 scores for the internal reports from center 1. Abbreviations: NIHSS, National Institutes of Health Stroke Scale. ASPECTS, Alberta Stroke Program Early CT Score. mTICI, modified Thrombolysis in Cerebral Infarction. ASA, acetylsalicylic acid. FDCT, flat detector computed tomography. ICH, intracranial hemorrhage.

| **Category** | **Precision Llama3.1 405B German prompt (%)** | **Recall Llama3.1 405B German prompt (%)** | **F1 Llama3.1 405B German prompt (%)** | **Precision Llama3 70B English prompt (%)** | **Recall Llama3 70B English prompt (%)** | **F1 Llama3 70B English prompt (%)** | **Precision Llama3 70B German prompt (%)** | **Recall Llama3 70B German prompt (%)** | **F1 Llama3 70B German prompt (%)** | **Precision Llama3 8B English prompt (%)** | **Recall Llama3 8B English prompt (%)** | **F1 Llama3 8B English prompt (%)** | **Precision Mixtral 8X7B English prompt (%)** | **Recall Mixtral 8X7B English prompt (%)** | **F1 Mixtral 8X7B English prompt (%)** |
| --- | --- | --- | --- | --- | --- | --- | --- | --- | --- | --- | --- | --- | --- | --- | --- |
| Date of intervention | 97.98 | 98.98 | 98.48 | 96.00 | 100.00 | 97.96 | 96.00 | 100.00 | 97.96 | 96.00 | 100.00 | 97.96 | 100.00 | 100.00 | 100.00 |
| Localisation of vessel occlusion | 90.00 | 100.00 | 94.74 | 79.00 | 100.00 | 88.27 | 86.00 | 100.00 | 92.47 | 68.00 | 100.00 | 80.95 | 75.00 | 100.00 | 85.71 |
| Side of vessel occlusion | 97.00 | 100.00 | 98.48 | 97.00 | 100.00 | 98.48 | 95.00 | 100.00 | 97.44 | 94.00 | 100.00 | 96.91 | 96.00 | 100.00 | 97.96 |
| NIHSS | 100.00 | 100.00 | 100.00 | 92.86 | 100.00 | 96.30 | 92.86 | 100.00 | 96.30 | 100.00 | 100.00 | 100.00 | 92.86 | 100.00 | 96.30 |
| ASPECTS | 100.00 | 100.00 | 100.00 | 100.00 | 100.00 | 100.00 | 100.00 | 100.00 | 100.00 | 98.04 | 100.00 | 99.01 | 100.00 | 96.00 | 97.96 |
| Intravenous thrombolysis | 100.00 | 100.00 | 100.00 | 27.63 | 91.30 | 42.42 | 74.19 | 100.00 | 85.19 | 69.57 | 69.57 | 69.57 | 100.00 | 91.30 | 95.45 |
| Symptom onset | 94.38 | 100.00 | 97.11 | 97.75 | 100.00 | 98.86 | 96.59 | 98.84 | 97.70 | 80.00 | 100.00 | 88.89 | 86.60 | 100.00 | 92.82 |
| Arrival at thrombectomy center | 78.13 | 100.00 | 87.72 | 80.00 | 96.00 | 87.27 | 79.03 | 98.00 | 87.50 | 64.10 | 100.00 | 78.13 | 52.87 | 100.00 | 69.17 |
| Stroke imaging | 94.38 | 100.00 | 97.11 | 89.77 | 98.75 | 94.05 | 88.64 | 98.73 | 93.41 | 83.53 | 92.21 | 87.65 | 66.30 | 100.00 | 79.74 |
| Groin puncture | 96.88 | 98.94 | 97.89 | 95.83 | 98.92 | 97.35 | 95.83 | 98.92 | 97.35 | 91.75 | 100.00 | 95.70 | 91.58 | 98.86 | 95.08 |
| First intracranial run | 98.80 | 100.00 | 99.39 | 92.05 | 100.00 | 95.86 | 95.35 | 100.00 | 97.62 | 88.51 | 98.72 | 93.33 | 83.91 | 97.33 | 90.12 |
| First thrombectomy maneuver | 98.85 | 98.85 | 98.85 | 96.63 | 98.85 | 97.73 | 98.85 | 98.85 | 98.85 | 87.50 | 100.00 | 93.33 | 91.30 | 100.00 | 95.45 |
| Last thrombectomy maneuver | 58.62 | 98.08 | 73.38 | 63.22 | 98.21 | 76.92 | 58.75 | 92.16 | 71.76 | 36.84 | 100.00 | 53.85 | 64.13 | 98.33 | 77.63 |
| Final run | 97.50 | 97.50 | 97.50 | 75.28 | 98.53 | 85.35 | 91.86 | 98.75 | 95.18 | 80.77 | 87.50 | 84.00 | 84.72 | 83.56 | 84.14 |
| Number of thrombectomy maneuvers | 94.68 | 93.68 | 94.18 | 92.47 | 92.47 | 92.47 | 94.57 | 91.58 | 93.05 | 87.76 | 97.73 | 92.47 | 90.91 | 98.90 | 94.74 |
| mTICI | 97.00 | 100.00 | 98.48 | 96.97 | 98.97 | 97.96 | 97.98 | 100.00 | 98.98 | 58.76 | 96.61 | 73.08 | 95.88 | 97.89 | 96.88 |
| Balloon guide catheter | 77.78 | 93.33 | 84.85 | 78.18 | 95.56 | 86.00 | 77.36 | 91.11 | 83.67 | 83.02 | 97.78 | 89.80 | 86.84 | 73.33 | 79.52 |
| Distal aspiration | 98.00 | 100.00 | 98.99 | 98.00 | 100.00 | 98.99 | 98.98 | 98.98 | 98.98 | 98.97 | 97.96 | 98.46 | 98.00 | 100.00 | 98.99 |
| Stentretriever | 100.00 | 100.00 | 100.00 | 100.00 | 98.94 | 99.47 | 100.00 | 100.00 | 100.00 | 94.95 | 100.00 | 97.41 | 100.00 | 100.00 | 100.00 |
| Extracranial stent | 100.00 | 88.89 | 94.12 | 100.00 | 55.56 | 71.43 | 100.00 | 55.56 | 71.43 | 0.00 | 0.00 | 0.00 | 100.00 | 33.33 | 50.00 |
| Intracranial stent | 66.67 | 100.00 | 80.00 | 33.33 | 100.00 | 50.00 | 18.18 | 100.00 | 30.77 | 22.22 | 100.00 | 36.36 | 28.57 | 100.00 | 44.44 |
| ASA | 100.00 | 66.67 | 80.00 | 75.00 | 100.00 | 85.71 | 69.23 | 100.00 | 81.82 | 9.18 | 100.00 | 16.82 | 9.28 | 100.00 | 16.98 |
| Clopidogrel | 0.00 | 0.00 | 0.00 | 0.00 | 0.00 | 0.00 | 0.00 | 0.00 | 0.00 | 0.00 | 0.00 | 0.00 | 0.00 | 0.00 | 0.00 |
| Ticagrelor | 0.00 | 0.00 | 0.00 | 0.00 | 0.00 | 0.00 | 0.00 | 0.00 | 0.00 | 0.00 | 0.00 | 0.00 | 0.00 | 0.00 | 0.00 |
| Tirofiban | 100.00 | 100.00 | 100.00 | 100.00 | 100.00 | 100.00 | 100.00 | 100.00 | 100.00 | 100.00 | 100.00 | 100.00 | 18.18 | 100.00 | 30.77 |
| Heparin | 100.00 | 97.44 | 98.70 | 100.00 | 98.72 | 99.35 | 100.00 | 98.72 | 99.35 | 78.00 | 100.00 | 87.64 | 78.00 | 100.00 | 87.64 |
| FDCT | 92.86 | 97.01 | 94.89 | 92.54 | 92.54 | 92.54 | 92.96 | 98.51 | 95.65 | 93.94 | 92.54 | 93.23 | 94.20 | 97.01 | 95.59 |
| ICH | 84.21 | 94.12 | 88.89 | 100.00 | 47.06 | 64.00 | 94.12 | 94.12 | 94.12 | 100.00 | 41.18 | 58.33 | 94.12 | 94.12 | 94.12 |
