# Supplementary material for: Llama 3.1 405B Is Comparable to GPT-4 for Extraction of Data from Thrombectomy Reports—A Step Towards Secure Data Extraction
Source: Clin Neuroradiol. 2025 Feb 25;35(3):495–510. doi: 10.1007/s00062-025-01500-z (PMC12454497; doi:10.1007/s00062-025-01500-z)
Supplement: Supplementary file 4 — Supplementary table S4. Precision, recall, and F1 scores for the external reports from center 2 [file 62_2025_1500_MOESM4_ESM.docx]

**Supplementary table S4.** Precision, recall, and F1 scores for the external reports from center 2. Abbreviations: NIHSS, National Institutes of Health Stroke Scale. ASPECTS, Alberta Stroke Program Early CT Score. mTICI, modified Thrombolysis in Cerebral Infarction. ASA, acetylsalicylic acid. FDCT, flat detector computed tomography. ICH, intracranial hemorrhage.

| **Category** | **Precision Llama3.1 405B German prompt (%)** | **Recall Llama3.1 405B German prompt (%)** | **F1 Llama3.1 405B German prompt (%)** | **Precision Llama3 70B English prompt (%)** | **Recall Llama3 70B English prompt (%)** | **F1 Llama3 70B English prompt (%)** | **Precision Llama3 70B German prompt (%)** | **Recall Llama3 70B German prompt (%)** | **F1 Llama3 70B German prompt (%)** | **Precision Llama3 8B English prompt (%)** | **Recall Llama3 8B English prompt (%)** | **F1 Llama3 8B English prompt (%)** | **Precision Mixtral 8X7B English prompt (%)** | **Recall Mixtral 8X7B English prompt (%)** | **F1 Mixtral 8X7B English prompt (%)** |
| --- | --- | --- | --- | --- | --- | --- | --- | --- | --- | --- | --- | --- | --- | --- | --- |
| Date of intervention | 100.00 | 100.00 | 100.00 | 100.00 | 100.00 | 100.00 | 100.00 | 100.00 | 100.00 | 100.00 | 100.00 | 100.00 | 100.00 | 100.00 | 100.00 |
| Localisation of vessel occlusion | 86.67 | 100.00 | 92.86 | 93.33 | 100.00 | 96.55 | 90.00 | 100.00 | 94.74 | 53.33 | 100.00 | 69.57 | 73.33 | 100.00 | 84.62 |
| Side of vessel occlusion | 96.67 | 100.00 | 98.31 | 83.33 | 100.00 | 90.91 | 80.00 | 100.00 | 88.89 | 73.33 | 100.00 | 84.62 | 96.67 | 100.00 | 98.31 |
| NIHSS | 100.00 | 100.00 | 100.00 | 100.00 | 100.00 | 100.00 | 100.00 | 100.00 | 100.00 | 100.00 | 100.00 | 100.00 | 100.00 | 100.00 | 100.00 |
| ASPECTS | 100.00 | 100.00 | 100.00 | 100.00 | 100.00 | 100.00 | 100.00 | 100.00 | 100.00 | 100.00 | 100.00 | 100.00 | 100.00 | 100.00 | 100.00 |
| Intravenous thrombolysis | 100.00 | 100.00 | 100.00 | 57.14 | 61.54 | 59.26 | 100.00 | 100.00 | 100.00 | 75.00 | 23.08 | 35.29 | 100.00 | 69.23 | 81.82 |
| Symptom onset | 100.00 | 100.00 | 100.00 | 70.83 | 100.00 | 82.93 | 89.47 | 100.00 | 94.44 | 53.33 | 100.00 | 69.57 | 53.33 | 100.00 | 69.57 |
| Arrival at thrombectomy center | 0.00 | 0.00 | 0.00 | 0.00 | 0.00 | 0.00 | 0.00 | 0.00 | 0.00 | 0.00 | 0.00 | 0.00 | 0.00 | 0.00 | 0.00 |
| Stroke imaging | 100.00 | 100.00 | 100.00 | 86.67 | 100.00 | 92.86 | 100.00 | 100.00 | 100.00 | 48.00 | 92.31 | 63.16 | 28.00 | 87.50 | 42.42 |
| Groin puncture | 44.83 | 100.00 | 61.90 | 43.33 | 100.00 | 60.47 | 43.33 | 100.00 | 60.47 | 44.83 | 100.00 | 61.90 | 40.00 | 100.00 | 57.14 |
| First intracranial run | 100.00 | 100.00 | 100.00 | 90.91 | 100.00 | 95.24 | 76.92 | 100.00 | 86.96 | 35.71 | 100.00 | 52.63 | 46.67 | 100.00 | 63.64 |
| First thrombectomy maneuver | 100.00 | 100.00 | 100.00 | 54.55 | 100.00 | 70.59 | 75.00 | 100.00 | 85.71 | 41.38 | 100.00 | 58.54 | 32.00 | 100.00 | 48.48 |
| Last thrombectomy maneuver | 0.00 | 0.00 | 0.00 | 0.00 | 0.00 | 0.00 | 0.00 | 0.00 | 0.00 | 0.00 | 0.00 | 0.00 | 6.25 | 50.00 | 11.11 |
| Final run | 90.00 | 100.00 | 94.74 | 53.33 | 100.00 | 69.57 | 83.33 | 100.00 | 90.91 | 55.56 | 100.00 | 71.43 | 85.71 | 66.67 | 75.00 |
| Number of thrombectomy maneuvers | 80.95 | 68.00 | 73.91 | 58.33 | 73.68 | 65.12 | 53.33 | 36.36 | 43.24 | 56.00 | 73.68 | 63.64 | 60.00 | 100.00 | 75.00 |
| mTICI | 96.55 | 100.00 | 98.25 | 93.33 | 100.00 | 96.55 | 93.33 | 100.00 | 96.55 | 53.57 | 93.75 | 68.18 | 92.86 | 96.30 | 94.55 |
| Balloon guide catheter | 0.00 | 0.00 | 0.00 | 0.00 | 0.00 | 0.00 | 0.00 | 0.00 | 0.00 | 0.00 | 0.00 | 0.00 | 0.00 | 0.00 | 0.00 |
| Distal aspiration | 96.67 | 100.00 | 98.31 | 96.67 | 100.00 | 98.31 | 96.67 | 100.00 | 98.31 | 96.67 | 100.00 | 98.31 | 96.67 | 100.00 | 98.31 |
| Stentretriever | 100.00 | 100.00 | 100.00 | 100.00 | 100.00 | 100.00 | 100.00 | 100.00 | 100.00 | 63.33 | 100.00 | 77.55 | 100.00 | 100.00 | 100.00 |
| Extracranial stent | 100.00 | 100.00 | 100.00 | 100.00 | 100.00 | 100.00 | 100.00 | 100.00 | 100.00 | 0.00 | 100.00 | 0.00 | 100.00 | 100.00 | 100.00 |
| Intracranial stent | 100.00 | 100.00 | 100.00 | 100.00 | 100.00 | 100.00 | 75.00 | 100.00 | 85.71 | 75.00 | 100.00 | 85.71 | 100.00 | 100.00 | 100.00 |
| ASA | 100.00 | 100.00 | 100.00 | 44.44 | 100.00 | 61.54 | 40.00 | 100.00 | 57.14 | 13.79 | 100.00 | 24.24 | 13.79 | 100.00 | 24.24 |
| Clopidogrel | 0.00 | 0.00 | 0.00 | 0.00 | 0.00 | 0.00 | 0.00 | 0.00 | 0.00 | 0.00 | 0.00 | 0.00 | 0.00 | 0.00 | 0.00 |
| Ticagrelor | 0.00 | 0.00 | 0.00 | 0.00 | 0.00 | 0.00 | 0.00 | 0.00 | 0.00 | 0.00 | 0.00 | 0.00 | 0.00 | 0.00 | 0.00 |
| Tirofiban | 100.00 | 100.00 | 100.00 | 100.00 | 100.00 | 100.00 | 100.00 | 100.00 | 100.00 | 100.00 | 100.00 | 100.00 | 28.57 | 100.00 | 44.44 |
| Heparin | 100.00 | 100.00 | 100.00 | 100.00 | 100.00 | 100.00 | 100.00 | 100.00 | 100.00 | 100.00 | 100.00 | 100.00 | 100.00 | 100.00 | 100.00 |
| FDCT | 0.00 | 0.00 | 0.00 | 0.00 | 0.00 | 0.00 | 0.00 | 0.00 | 0.00 | 0.00 | 0.00 | 0.00 | 0.00 | 0.00 | 0.00 |
| ICH | 100.00 | 100.00 | 100.00 | 100.00 | 100.00 | 100.00 | 100.00 | 100.00 | 100.00 | 100.00 | 100.00 | 100.00 | 100.00 | 50.00 | 66.67 |
