# Supplementary material for: Llama 3.1 405B Is Comparable to GPT-4 for Extraction of Data from Thrombectomy Reports—A Step Towards Secure Data Extraction
Source: Clin Neuroradiol. 2025 Feb 25;35(3):495–510. doi: 10.1007/s00062-025-01500-z (PMC12454497; doi:10.1007/s00062-025-01500-z)
Supplement: Supplementary file 5 — Table S5. Cohen’s kappa of the different models against the neuroradiologist by category [file 62_2025_1500_MOESM5_ESM.docx]

**Table S5.** Cohen's kappa of the different models against the neuroradiologist by category. Abbreviations: NIHSS, National Institutes of Health Stroke Scale. ASPECTS, Alberta Stroke Program Early CT Score. mTICI, modified Thrombolysis in Cerebral Infarction. BGC, balloon guide catheter. ASA, acetylsalicylic acid. FPCT, flat panel computed tomography. ICH, intracranial hemorrhage. NaN, Not a Number

|  | **Llama 3.1 405B German prompt** | |  |  | **Llama 3 70B English prompt** | |  |  | **Llama 3 70B German prompt** | | |  | **Llama 3 8B English prompt** | | |  | **Mixtral 8X7B English prompt** | | |  |
| --- | --- | --- | --- | --- | --- | --- | --- | --- | --- | --- | --- | --- | --- | --- | --- | --- | --- | --- | --- | --- |
| **Category** | **Kappa internal reports** | **P internal reports** | **Kappa external reports** | **P external reports** | **Kappa internal reports** | **P internal reports** | **Kappa external reports** | **P external reports** | **Kappa internal reports** | **P internal reports** | **Kappa external reports** | **P external reports** | **Kappa internal reports** | **P internal reports** | **Kappa external reports** | **P external reports** | **Kappa internal reports** | **P internal reports** | **Kappa external reports** | **P external reports** |
| Date of Intervention | 0.97 | <.001 | 1.00 | <.001 | 0.96 | <.001 | 1.00 | <.001 | 0.96 | <.001 | 1.00 | <.001 | 0.96 | <.001 | 1.00 | <.001 | 1.00 | <.001 | 1.00 | <.001 |
| Location of vessel occlusion | 0.85 | <.001 | 0.76 | <.001 | 0.70 | <.001 | 0.87 | <.001 | 0.80 | <.001 | 0.81 | <.001 | 0.56 | <.001 | 0.33 | <.001 | 0.64 | <.001 | 0.58 | <.001 |
| Side of vessel occlusion | 0.95 | <.001 | 0.95 | <.001 | 0.95 | <.001 | 0.74 | <.001 | 0.91 | <.001 | 0.68 | <.001 | 0.89 | <.001 | 0.58 | <.001 | 0.93 | <.001 | 0.95 | <.001 |
| NIHSS | 1.00 | <.001 | 1.00 | <.001 | 0.96 | <.001 | 1.00 | <.001 | 0.96 | <.001 | 1.00 | <.001 | 1.00 | <.001 | 1.00 | <.001 | 0.96 | <.001 | 1.00 | <.001 |
| ASPECTS | 1.00 | <.001 | 1.00 | <.001 | 1.00 | <.001 | 1.00 | <.001 | 1.00 | <.001 | 1.00 | <.001 | 0.99 | <.001 | 1.00 | <.001 | 0.97 | <.001 | 1.00 | <.001 |
| Intravenous Thrombolysis | 0.95 | <.001 | 0.84 | <.001 | 0.25 | <.001 | 0.35 | 0.005 | 0.29 | <.001 | 0.63 | <.001 | 0.48 | <.001 | 0.10 | 0.14 | 0.95 | <.001 | 0.54 | <.001 |
| Symptom onset | 0.94 | <.001 | 1.00 | <.001 | 0.98 | <.001 | 0.72 | <.001 | 0.95 | <.001 | 0.92 | <.001 | 0.77 | <.001 | 0.48 | <.001 | 0.85 | <.001 | 0.48 | <.001 |
| Arrival at thrombectomy center | 0.83 | <.001 | NaN | NaN | 0.82 | <.001 | NaN | NaN | 0.83 | <.001 | NaN | NaN | 0.68 | <.001 | 0.00 | NaN | 0.56 | <.001 | 0.00 | NaN |
| Time of stroke imaging | 0.95 | <.001 | 1.00 | <.001 | 0.90 | <.001 | 0.91 | <.001 | 0.89 | <.001 | 1.00 | <.001 | 0.80 | <.001 | 0.48 | <.001 | 0.68 | <.001 | 0.29 | <.001 |
| Groin puncture time | 0.96 | <.001 | 0.45 | <.001 | 0.95 | <.001 | 0.42 | <.001 | 0.95 | <.001 | 0.42 | <.001 | 0.92 | <.001 | 0.45 | <.001 | 0.91 | <.001 | 0.44 | <.001 |
| Time of first intracranial run | 0.99 | <.001 | 1.00 | <.001 | 0.93 | <.001 | 0.94 | <.001 | 0.96 | <.001 | 0.84 | <.001 | 0.89 | <.001 | 0.36 | <.001 | 0.83 | <.001 | 0.59 | <.001 |
| Time of first thrombectomy maneuver | 0.98 | <.001 | 1.00 | <.001 | 0.96 | <.001 | 0.60 | <.001 | 0.98 | <.001 | 0.81 | <.001 | 0.88 | <.001 | 0.41 | <.001 | 0.92 | <.001 | 0.36 | <.001 |
| Time of last thrombectomy maneuver | 0.61 | <.001 | 0.26 | <.001 | 0.65 | <.001 | 0.10 | 0.04 | 0.60 | <.001 | 0.22 | 0.005 | 0.39 | <.001 | 0.09 | 0.05 | 0.65 | <.001 | 0.15 | 0.01 |
| Final run | 0.96 | <.001 | 0.94 | <.001 | 0.76 | <.001 | 0.65 | <.001 | 0.92 | <.001 | 0.89 | <.001 | 0.75 | <.001 | 0.63 | <.001 | 0.75 | <.001 | 0.72 | <.001 |
| Number of thrombectomy maneuvers | 0.86 | <.001 | 0.49 | <.001 | 0.82 | <.001 | 0.35 | <.001 | 0.84 | <.001 | 0.18 | 0.004 | 0.82 | <.001 | 0.32 | <.001 | 0.87 | <.001 | 0.45 | <.001 |
| mTICI | 0.95 | <.001 | 0.95 | <.001 | 0.94 | <.001 | 0.90 | <.001 | 0.97 | <.001 | 0.89 | <.001 | 0.47 | <.001 | 0.44 | <.001 | 0.91 | <.001 | 0.84 | <.001 |
| BGC | 0.70 | <.001 | 0.00 | NaN | 0.72 | <.001 | 0.00 | NaN | 0.68 | <.001 | 0.00 | NaN | 0.80 | <.001 | 0.00 | NaN | 0.65 | <.001 | 0.00 | NaN |
| Use of distal aspiration | 0.00 | NaN | 0.00 | 1 | 0.00 | NaN | 0.00 | 1 | 0.49 | <.001 | 0.00 | 1 | 0.39 | <.001 | 0.00 | 1 | 0.00 | NaN | 0.00 | 1 |
| Use of stentretriever | 1.00 | <.001 | 1.00 | <.001 | 0.92 | <.001 | 1.00 | <.001 | 1.00 | <.001 | 1.00 | <.001 | 0.27 | <.001 | 0.00 | NaN | 1.00 | <.001 | 1.00 | <.001 |
| Extracranial stent implanted | 0.94 | <.001 | 1.00 | <.001 | 0.69 | <.001 | 1.00 | <.001 | 0.69 | <.001 | 1.00 | <.001 | 0.00 | 1 | 1.00 | <.001 | 0.48 | <.001 | 1.00 | <.001 |
| Intracranial stent implanted | 0.23 | <.001 | 0.84 | <.001 | 0.48 | <.001 | 1.00 | <.001 | 0.28 | <.001 | 0.84 | <.001 | 0.34 | <.001 | 0.84 | <.001 | 0.43 | <.001 | 1.00 | <.001 |
| ASA | 0.54 | <.001 | 0.87 | <.001 | 0.84 | <.001 | 0.53 | 0.001 | 0.38 | <.001 | 0.47 | 0.002 | 0.00 | 0.65 | 0.01 | 0.69 | 0.01 | 0.58 | 0.01 | 0.69 |
| Clopidogrel | 0.00 | 1 | 0.00 | 1 | 0.00 | NaN | 0.00 | NaN | 0.00 | NaN | 0.00 | NaN | 0.00 | NaN | 0.00 | NaN | NaN | NaN | 0.00 | NaN |
| Ticagrelor | 0.00 | NaN | NaN | NaN | 0.00 | 1 | NaN | NaN | 0.00 | NaN | NaN | NaN | 0.00 | 1 | NaN | NaN | 0.00 | NaN | NaN | NaN |
| Tirofiban | 0.56 | <.001 | 1.00 | <.001 | 1.00 | <.001 | 1.00 | <.001 | 0.16 | <.001 | 1.00 | <.001 | 1.00 | <.001 | 1.00 | <.001 | 0.28 | <.001 | 0.38 | 0.008 |
| Heparin | 0.92 | <.001 | NaN | NaN | 0.97 | <.001 | NaN | NaN | 0.51 | <.001 | NaN | NaN | 0.00 | NaN | NaN | NaN | 0.00 | NaN | NaN | NaN |
| FDCT | 0.84 | <.001 | 0.00 | NaN | 0.77 | <.001 | 0.00 | NaN | 0.86 | <.001 | 0.00 | NaN | 0.80 | <.001 | 0.00 | NaN | 0.86 | <.001 | 0.00 | NaN |
| ICH | 0.84 | <.001 | 1.00 | <.001 | 0.60 | <.001 | 1.00 | <.001 | 0.93 | <.001 | 1.00 | <.001 | 0.54 | <.001 | 1.00 | <.001 | 0.93 | <.001 | 0.65 | <.001 |
